# Oscillometry for personalizing continuous distending pressure maneuvers: an observational study in extremely preterm infants

**DOI:** 10.1186/s12931-023-02639-4

**Published:** 2024-01-04

**Authors:** Chiara Veneroni, Raffaele L. Dellacà, Erik Küng, Beatrice Bonomi, Angelika Berger, Tobias Werther

**Affiliations:** 1 TechRes Lab, Department of Electronics, Information and Biomedical Engineering (DEIB), Politecnico di Milano University, Via G. Colombo 40, Milan, 20133 Italy; 2 Division of Neonatology, Pediatric Intensive Care and Neuropediatrics, Department of Pediatrics, Comprehensive Center for Pediatrics, Medical University of Vienna, Vienna, Austria

**Keywords:** Forced oscillation technique, Mechanical ventilation, Ventilation-induced lung injury, Lung mechanics, High-frequency oscillatory ventilation

## Abstract

**Rationale:**

Lung recruitment and continuous distending pressure (CDP) titration are critical for assuring the efficacy of high-frequency ventilation (HFOV) in preterm infants. The limitation of oxygenation (peripheral oxygen saturation, SpO_2_) in optimizing CDP calls for evaluating other non-invasive bedside measurements. Respiratory reactance (Xrs) at 10 Hz measured by oscillometry reflects lung volume recruitment and tissue strain. In particular, lung volume recruitment and decreased tissue strain result in increased Xrs values.

**Objectives:**

In extremely preterm infants treated with HFOV as first intention, we aimed to measure the relationship between CDP and Xrs during SpO_2_-driven CDP optimization.

**Methods:**

In this prospective observational study, extremely preterm infants born before 28 weeks of gestation undergoing SpO_2_-guided lung recruitment maneuvers were included in the study. SpO_2_ and Xrs were recorded at each CDP step. The optimal CDP identified by oxygenation (CDP_Opt_SpO2_) was compared to the CDP providing maximal Xrs on the deflation limb of the recruitment maneuver (CDP_Xrs_).

**Results:**

We studied 40 infants (gestational age at birth = 22^+ 6^-27^+ 5^ wk; postnatal age = 1–23 days). Measurements were well tolerated and provided reliable results in 96% of cases. On average, Xrs decreased during the inflation limb and increased during the deflation limb. Xrs changes were heterogeneous among the infants for the amount of decrease with increasing CDP, the decrease at the lowest CDP of the deflation limb, and the hysteresis of the Xrs vs. CDP curve. In all but five infants, the hysteresis of the Xrs vs. CDP curve suggested effective lung recruitment. CDP_Opt_SpO2_ and CDP_Xrs_ were highly correlated (ρ = 0.71, p < 0.001) and not statistically different (median difference [range] = -1 [-3; 9] cmH_2_O). However, CDP_Xrs_ were equal to CDP_Opt_SpO2_ in only 6 infants, greater than CDP_Opt_SpO2_ in 10, and lower in 24 infants.

**Conclusions:**

The Xrs changes described provide complementary information to oxygenation. Further investigation is warranted to refine recruitment maneuvers and CPD settings in preterm infants.

**Supplementary Information:**

The online version contains supplementary material available at 10.1186/s12931-023-02639-4.

## Introduction

Extremely preterm infants often need mechanical ventilation in their first days after birth [1]. Due to the preterm infant’s immature lungs, lung protective ventilation strategies are used in an attempt to limit ventilator-induced lung injury. A lung protective ventilation strategy minimizes alveolar overdistention during inspiration, reverses atelectasis, and stabilizes open lung units [2].

High-frequency oscillatory ventilation (HFOV) is an established ventilation mode in preterm infants associated with minimal tidal volumes and pressure changes at the alveoli [3]. Its efficacy critically depends on optimizing and stabilizing lung volume [4]. To this aim, periodically repeated lung recruitment maneuvers and continuous distending pressure (CDP) optimization are advocated. The strategy of lung recruitment, however, is not well defined. In clinical practice, oxygen response is widely used as a surrogate for lung volume [5]. In an animal study, recruitment maneuvers, consisting of stepwise CDP increases and decreases, were shown beneficial for oxygenation [6]. This strategy was adopted in a seminal study on HFOV initiation in preterm infants with RDS [7]. These maneuvers have a low risk of lung hyperinflation, air-leak syndrome, critically worsening cardiac function, and cerebral adverse events (e.g., intraventricular hemorrhage) in preterm infants [8, 9]. However, the heterogeneity of infants’ responses to recruitment maneuvers is well documented. Therefore, methods for evaluating the respiratory system response to pressure changes and identifying the optimal CDP are required [7]. Currently, recruitment maneuvers and optimal CDP identification are driven by oxygenation determined by peripheral oxygen saturation (SpO_2_) corrected by the fraction of inspired oxygen (FiO_2_) [3]. SpO_2_ is easily available at the bedside and correlates with lung volumes [10, 11]. However, it is only an indirect marker of lung volume recruitment and is affected by various factors, including hemodynamic changes and pulmonary hypertension [12]. Moreover, difficulties in determining SpO_2_ target ranges and FiO_2_ thresholds, together with the variable infants’ capabilities of increasing their spontaneous breathing activity to maintain SpO_2_ in the target range, further complicate using SpO_2_/FiO_2_ for CDP titration. Also, a previous study showed that the efficacy of the recruitment maneuver evaluated on changes in end-expiratory lung volume (monitored by respiratory inductive plethysmography) rather than SpO_2_ correlated better with improvements in clinical condition after 1 h [13].

Oscillometry assesses respiratory reactance (Xrs) by measuring pressure and flow at the airways opening while a small-amplitude, high-frequency pressure stimulus is superimposed to ventilation. Xrs expresses the inertial and elastic properties of the respiratory system, reflecting lung volume recruitment and tissue strain. In particular, Xrs decreases as a result of lung volume derecruitment and/or lung (over)distension [14], similar to lung compliance. Oscillometry has recently become available at the bedside for newborns and has been safely applied to preterm infants during mechanical ventilation [15–17].

In preterm infants treated with HFOV as first intention, we aimed to measure the relationship between CDP, SpO_2_/FiO_2_, and Xrs during SpO_2_-driven CDP optimization.

## Methods

This was a prospective observational study and sub-trial of a randomized controlled trial (ClinicalTrials.gov ID: NCT04289324) on recruitment maneuver during HFOV comparing infants receiving recruitment maneuvers either at 12 h intervals and when clinically indicated (intervention) or only when clinically indicated (control) [18]. The study was conducted at the neonatal intensive care unit of the Medical University of Vienna, Vienna, Austria, between March 2020 and June 2022. The study was approved by the local ethics committee (EK 1161/2019).

### Study population

Preterm infants born before 28 weeks of gestation without any congenital anomalies of the heart and/or the lungs (as reported in ultrasound and/or fetal magnetic resonance imaging) were eligible in their first four weeks of postnatal age. Infants on HFOV were enrolled upon the availability of the study team to perform measurements during a single lung recruitment maneuver performed during the randomized controlled trial mentioned above. Written informed consent from parents or legal guardians was obtained before performing the measurements.

### Study protocol

SpO_2_, transcutaneous CO_2_ (TcCO_2_), heart rate, and invasive blood pressure were continuously monitored as per local standard of care. Starting at the CDP in use (initial CDP, CDP_i_), CDP was increased (inflation limb) approximatively every 5 min (allowing intervals of 2–15 min upon the decision of the caregiving team) by 2 cmH_2_O (allowing for steps of 1 cmH_2_O when CDP was greater than 20 cmH_2_O). The fraction of inspired oxygen (FiO_2_) was reduced stepwise, keeping SpO_2_ within the predefined target range (88–96% or 90–96% in the presence of pulmonary hypertension requiring medication). The inflation limb ended when SpO_2_ no longer improved or FiO_2_ was ≤ 0.25. From the maximal CDP (CDP_max_), CDP was gradually decreased (deflation limb) approximately every 5 min (allowing intervals of 2–15 min upon the decision of the caregiving team) by 2 cmH_2_O (allowing for steps of 1 cmH_2_O when CDP was lower than the initial CDP) until either a sustained SpO_2_ drop of at least 5% or a SpO_2_ value below 88% indicated the reaching of the closing CDP (minimal CDP of the recruitment maneuver, CDP_min_). The minimum allowed CDP was 5 cmH_2_O. The HFOV frequency was kept at the clinically set values. The pressure amplitude was adjusted to target TcCO_2_ between 40 and 60 mmHg (SenTec Digital Monitor, Therwil, Switzerland, with a probe temperature of 41 °C). CDP_Opt_SpO2_ was defined as CDP_min_ +1 or 2 cmH_2_O [7]. Before setting CDP_Opt_SpO2_ on the ventilator (final CPD, CDP_f_) CDP_max_ was set for approximately 5 min (re-recruitment after the deflation limb) to promote lung volume re-recruitment after the application of CDP_min_. Recruitment maneuvers were advised after the following situations: change of position (from prone to supine or vice versa), any manipulation with FiO_2_ increase of 0.1 or SpO_2_ decrease > 10% for > 5 min (e.g., suctioning, endotracheal tube disconnection), surfactant application, and suspected or confirmed atelectasis (e.g., diagnosed on chest X-ray). Infants were not disconnected from the ventilator nor underwent suctioning during the maneuvers, as these would have modified the lung volume history and the maneuver efficacy. The infant’s head and body position were not changed during the recruitment maneuver.

### Measurements

SpO_2_ (Covidien-Nellcor, Boulder, CO) was monitored continuously and recorded at the end of each CDP step. Conversely, Xrs was measured after 3 min of stabilization at each CDP step (or at the end of the CDP step for shorter steps). The ventilator computed Xrs values (Fabian FOT 150,204 V5.0, Vyaire srl, US). When requested by the clinician, the ventilator reduced HFOV amplitude for ~ 3 s and set the oscillation frequency to 10 Hz, keeping the same CDP. At each time point, the ventilator provided a single measure as the mean Xrs values over the measurement time if the test passed a quality check for excluding measurements at risk of being affected by artifacts. No specific calibration procedure was required for performing the measurements. Clinicians were blinded to Xrs data. In two infants, we repeated oscillometry also at 6 min after CDP change to evaluate the stability of the Xrs values with time.

### Data analysis

The oxygen saturation index (OSI) was calculated as CDP × FiO_2_ × 100 ÷ SpO_2_. Xrs values at each CDP were exported from the ventilator and corrected for the impedance of the endotracheal tube (considering the diameter and the actual length of the tube) [19]. Missing Xrs data were estimated by linear interpolating Xrs values at the previous and following CDP steps. The average Xrs and SpO_2_/FiO_2_ vs. CDP relationship was computed by normalizing CDP between 0% (CDP_min_) and 100% (CDP_max_) and linearly interpolating the measured data to obtain values each 10% or 20%. CDP_Xrs_ was defined as the one providing maximal Xrs on the deflation limb of the recruitment. Xrs and SpO_2_/FiO_2_ values were averaged at CDP_i_, CDP_max_, CDP_min_, CDP_Opt_SpO2_, and CDP_Xrs_. The difference between CDP_Xrs_ and CDP_Opt_SpO2_ was computed with a resolution of ± 1 cmH_2_O as CDP steps of the deflation limb were mainly of 2 cmH_2_O.

To quantify Xrs changes with CDP, we defined the following parameters: (1) ΔX_inf_limb_ as the difference in Xrs at CDP_i_ and CDP_max_ to evaluate the impact of increasing CDP; (2) ΔX_cl_ as the difference in Xrs at CDP_Xrs_ and CDP_min_ to evaluate the impact of the lowest CDP applied; (3) ΔX_rec_ as the difference in Xrs at CDP_Xrs_ during the deflation and the inflation limb. When CDP_Xrs_ was lower than CDP_i_, ΔX_rec_ was computed at CDP_i_ to guarantee that Xrs was compared at the same distending pressure. ΔX_rec_ evaluated the Xrs vs. CDP curve hysteresis associated with lung recruitment. We considered Xrs changes relevant only if higher than 6.3 cmH_2_O*s/L and 10% of its value. The reproducibility of Xrs measures in preterm infants was 6.3 cmH_2_O*s/L and was within 10% of its value for Xrs < − 60 cmH_2_O*s/L [20].

To understand whether the Xrs response to pressure increases during the inflation limb was predictive of post-maneuver overall lung volume recruitment (as assessed by ΔX_rec_), we evaluated the shape of the Xrs vs. CDP graphs during inflation limb searching for different patterns and performed an exploratory sub-group analysis (see Additional File 1).

### Statistical analysis

Friedman Repeated Measures Analysis of Variance on Ranks, followed by Pairwise Multiple Comparison by Tukey Test, as appropriate for data distribution, was used for comparing data at more than two CDPs. Correlations between variables were tested by the Spearman test. Differences were considered statistically significant for p < 0.05.

## Results

We studied 40 infants with heterogeneous demographic characteristics (Table 1). The CDP applied, the number of CDP steps, and the duration of CDP intervals varied among SpO_2_-driven CDP optimization (Table 1). In each infant, monitoring parameters could be maintained in individually predefined target ranges as per local standard of care throughout the recruitment maneuver. A total of 588 Xrs measurements were attempted. Seven infants missed Xrs values at some CDPs for a total of 14 missing Xrs measures (2% of the total measures) because the ventilator reported unreliable conditions to perform the measurements. Eleven infants presented inconsistent Xrs values at some CDP steps for a total of 16 Xrs values (3% of the successful measurements), as they were outliers of the depicted Xrs vs. CDP curve as judged by visual inspection.

**Table 1:** Patient and maneuver characteristics

GA at birth, weeks	24^+ 0^ [22^+ 6^ – 27^+ 5^]
Birth weight, g	612 [410–920]
Female	15 (37.5%)
Antenatal steroids:	
*Incomplete*	15 (37.5%)
*Complete*	23 (57.5%)
Postnatal age, days	4 [1–23]
Weight, g	701 [435–990]
Supine	31 (77.5%)
Receiving muscle relaxant	12 (30%)
Indication for recruitment maneuver:	
*After surfactant*	1 (2.5%)
*HFOV start*	4 (10%)
*Position change*	15 (37.5%)
*SpO*_*2*_*/FiO*_*2*_*drop*	4 (10%)
*Atelectasis*	7 (17.5%)
*As per protocol in the previous study (12 h intervals)*	9 (22.5%)
CDP step duration, mm:ss	6:10 [1:33–15:32]
Number of steps	14 [9–20]
Maneuver duration, min	92 [51–135]
CDP_i_, cmH_2_O	10.5 [7–13]
CDP_max_, cmH_2_O	20 [16–24]
CDP_min_, cmH_2_O	8 [5–13]
BPD:	
*Dead no BPD*	7 (17.5%)
*No BPD*	8 (20%)
*BPD_I*	10 (25%)
*BPD_II*	7 (17.5%)
*BPD_III*	6 (15%)
*BPD_IIIA*	2 (5%)

Data are reported as median [range] or number (percentage)

GA = Gestational Age; HFOV = High-frequency oscillatory ventilation; CDP = continuous distending pressure; CDP_i_ = initial CDP of the maneuver; CDP_max_ = maximal CDP of the maneuver; CDP_min_ = minimal CDP of the maneuver; BPD = Bronchopulmonary Dysplasia. BPD_I, BPD_II, BPD_III, BPD_IIIA = BPD grades as defined by Higgins et al. [31]

**Fig. 1:**
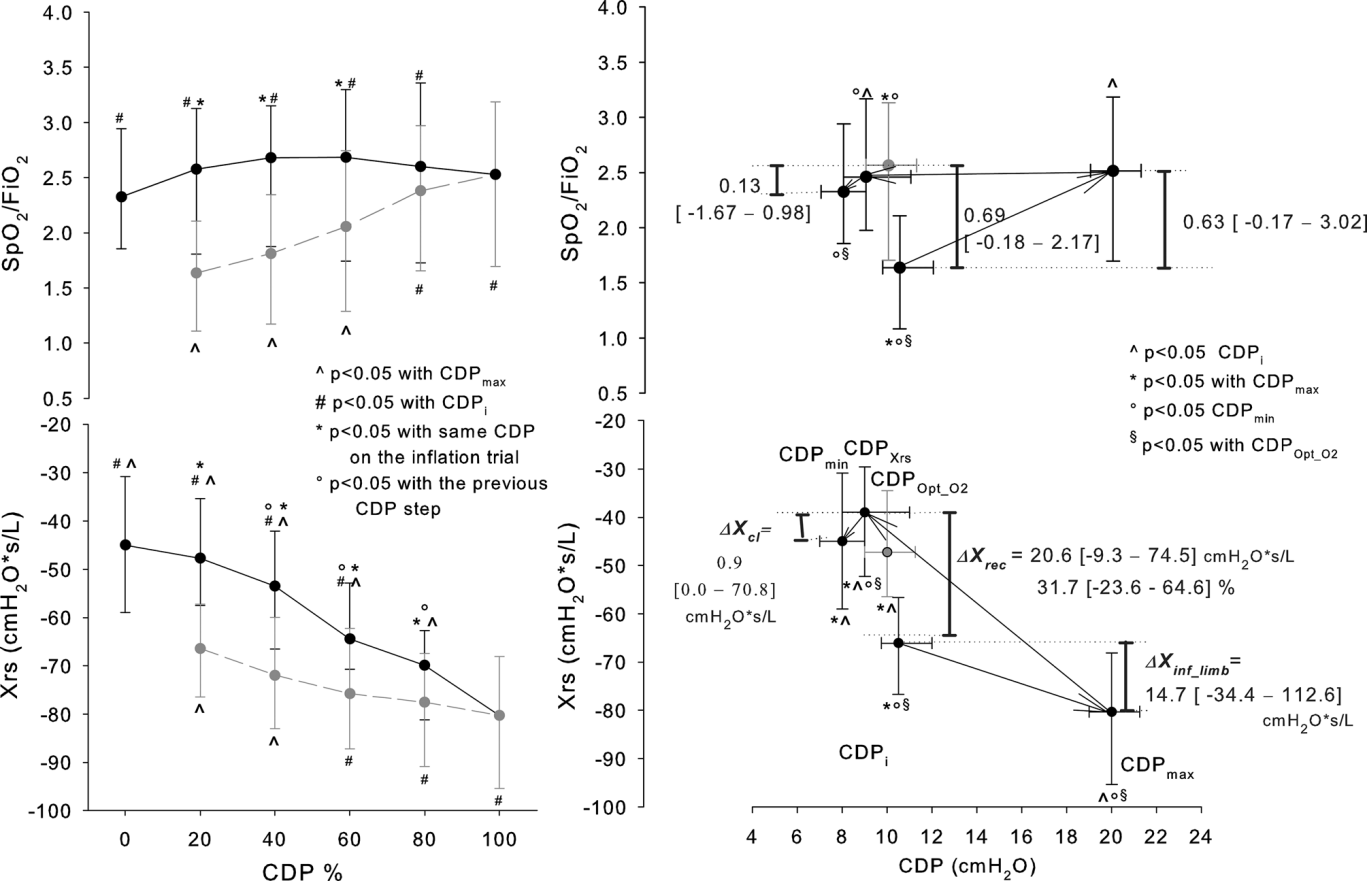
Median (IQR) SpO_2_/FiO_2_ (Upper Panels) and Xrs (Lower Panels) vs. CDP. *Left panels*: CDP was normalized between 0% (CDP_min_= minimal CDP) and 100% (CDP_max_= maximal CDP). Parameters are computed for each 20% CDP change by linear interpolating the measured data. The grey and black lines represent the inflation and the deflation limbs, respectively. *Right panels*: values are averaged at initial CDP (CDP_i_), maximal CDP (CDP_max_), CDP corresponding to maximal Xrs (CDP_Xrs_), minimal CDP (CDP_min_), optimal CDP as identified by SpO_2_ (CDP_Opt_O2_, grey circle). The figure reports the median [range] of Xrs and SpO_2_/FiO_2_ changes at selected CDPs. ΔX_inf_limb_ = the difference in Xrs between CDP_i_ and CDP_max_, evaluating the impact of increasing CDP. The difference between CDP_max_ and CDP_i_ was 10 [4–14] cmH_2_O and the Xrs change per cmH_2_O resulted: ΔX_inf_limb_/( CDP_max_ – CDP_i_ ) = 1.5 [-7.3–14.1] s/L. ΔX_cl_ = the difference in Xrs between CDP_Xrs_ and CDP_min_ to evaluate the impact of the lowest CDP applied. The difference between CDP_Xrs_ and CDP_min_ was 1 [0–10] cmH_2_O and the Xrs change per cmH_2_O resulted: ΔX_cl_/( CDP_Xrs_ - CDP_min_) = 0.2 [0–16.4] s/L. ΔX_rec_ = the difference in Xrs between CDP_Xrs_ during the inflation and the deflation limb to evaluate the hysteresis of the curve. It is reported as an absolute difference (cmH_2_O*s/L) and as a percentage of the value on the inflation limb. In 20 infants CDP_Xrs_ was lower than the CDP_i_, ΔX_rec_ was computed at CDP_i_

Initial Xrs values ranged from − 140 to -27 cmH_2_O*s/L and correlated with GA (ρ = 0.33, p = 0.04) but not SpO_2_/FiO_2_. In general, Xrs significantly changed with CDP from a minimum change of 13 cmH_2_O*s/L to a maximum change of 88 cmH_2_O*s/L. Xrs changes were heterogeneous among the infants for the amount of decrease at increasing CDP, the decrease at the lowest CDPs, and the hysteresis of Xrs vs. CDP curve (Fig. 1 right panel).

### Xrs vs. CPD curve: Xrs changes during the inflation limb and curve hysteresis

**Fig. 2:**
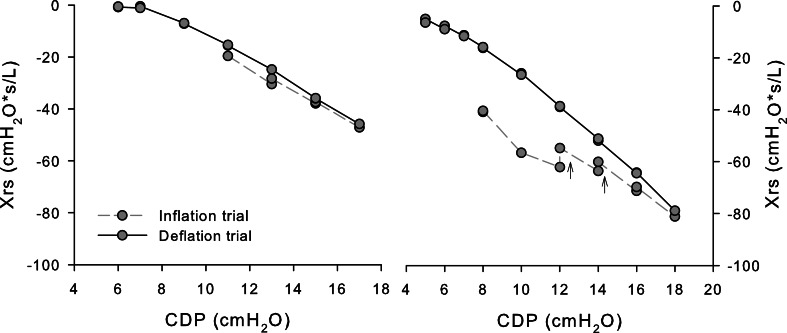
Xrs vs. CDP in two subjects with Xrs measured at 3 and 6 min after CDP change. Arrows indicate the temporal sequence of the measures

On average, Xrs decreased during the inflation limb and increased during the deflation limb (Fig. 1, left panel). However, in 6 infants, Xrs mainly increased with increasing CDP (ΔX_inf_limb_< 0). In all but 5 infants (87.5%), ΔX_rec_ was higher than the reproducibility of the Xrs measure. ΔX_rec_ was not dependent on the indication for performing the recruitment maneuver. Also, ΔX_rec_ was not entirely predictable from Xrs changes during the inflation limb. In fact, ΔX_rec_ weakly correlated to ΔX_inf_limb_ (ρ = 0.33, p = 0.04), and it was not possible to predict ΔX_rec_ higher than the reproducibility of Xrs measures from the pattern of Xrs vs. CDP graphs of the inflation limb. All the graphs showing Xrs increases or Xrs stability with increasing CDP for at least two CDP steps resulted in ΔX_rec_ higher than the Xrs reproducibility. However, graphs showing a continuous decrease in Xrs resulted in ΔX_rec_ both higher or lower than the Xrs reproducibility (Additional File 1: Figure S1). Repeated measures at the same CDP step showed Xrs values that were still increasing 3 min after the CDP increment in one infant at a few steps of the inflation limb (Fig. 2).

### Xrs vs. CPD curve: last steps of the deflation limb and re-recruitment after the maneuver

In 22 infants (55%), Xrs decreased during the last steps of the deflation limb (ΔX_cl_ > 0), and in 15 (37.5%), the decrease is higher than the reproducibility of the Xrs measure (ΔX_cl_ > 6.3 cmH_2_O*s/L and 10%).

There were no differences between Xrs and SpO_2_/FiO_2_ values at the CDP_Opt_SpO2_ of the deflation limb and after the re-recruitment step (at CDP_f_) (median difference of -1.2 cmH_2_O*s/L and − 0.04, respectively) (Table 2). The re-recruitment allowed Xrs to recover at least its value of the deflation limb at the corresponding CDP (within the reproducibility of the Xrs measure) in 34 infants (85%). In six of these infants, Xrs was even higher than the one during the deflation limb (median [range] of the change, 10.2 [7.0;15.9] cmH_2_O*s/L). In the remaining six infants, Xrs after the second fast recruitment was lower than its value of the deflation limb at the corresponding CDP (median [range] of the change − 10.7 [-24.6;- 10.3] cmH_2_O*s/L).

**Table 2:** Respiratory parameters at selected CDP

	CDP_i_	CDP_Xrs_	CDP_Opt_SpO2_	CDP_f_
**CDP**, cmH_2_O	10.5 (9.5; 12.0) [7–13]	9.0 (8.0; 11.0) [5–20]	10.0 (9.0; 11.5) [7–14]	10.0 (9.0; 11.5) [7–14]
**SpO**_**2**_ **/FiO**_**2**_	1.64 (1.08; 2.15) [0.86–3.46]	2.46 (1.95; 3.17)* [0.98–4.24]	2.56 (1.69; 3.13)* [1.04–4.38]	2.57 (1.95; 3.13)* [1.11–4.48]
**FiO**_**2**_	55 (42; 82) [26–100]	37 (30; 50)* [21–90]	35 (30; 54)* [21–90]	35 (30; 47)* [21–85]
**Xrs**, cmH_2_O*s/L	-66.1 (-76.7; -56.5) [-140.2 – -27.2]	-39.0 (-52.9; -29.3)* [-82.8 – -11.8]	-47.2 (-56.5; -33.8)*° [-99.7 – -18.5]	-47.7 (-61.3; -35.9)*° [-93.4 – -13.4]
**OSI**, cmH_2_O	6.77 (5.05; 8.86) [2.90–13.95]	3.94 (3.15; 5.47)* [3.15–5.47]	4.22 (3.23; 5.54)* [3.23–5.54]	4.27 (3.30; 5.64)* [2.01 – 9.04]

CDP_i_ = initial CDP of the maneuver; CDP_f_ = final CDP at the end of the procedure, after re-recruitment after the deflation limb; CDP_Opt_SpO2_ = optimal CDP as identified by oxygenation; CDP_Xrs_ = the CDP providing maximal Xrs on the deflation limb. Data are reported as median (interquartile range) [range]

* p < 0.05 with CDP_i_; ° p < 0.05 with CDP_Xrs_.

### Xrs and oxygenation changes

On average, and differently from Xrs, SpO_2_/FiO_2_ improved during the inflation limb and worsened at the deflation limb’s end. Changes in SpO_2_/FiO_2_ during the maneuver weakly correlated with Xrs (ρ = 0.28; p < 0.001).

**Fig. 3:**
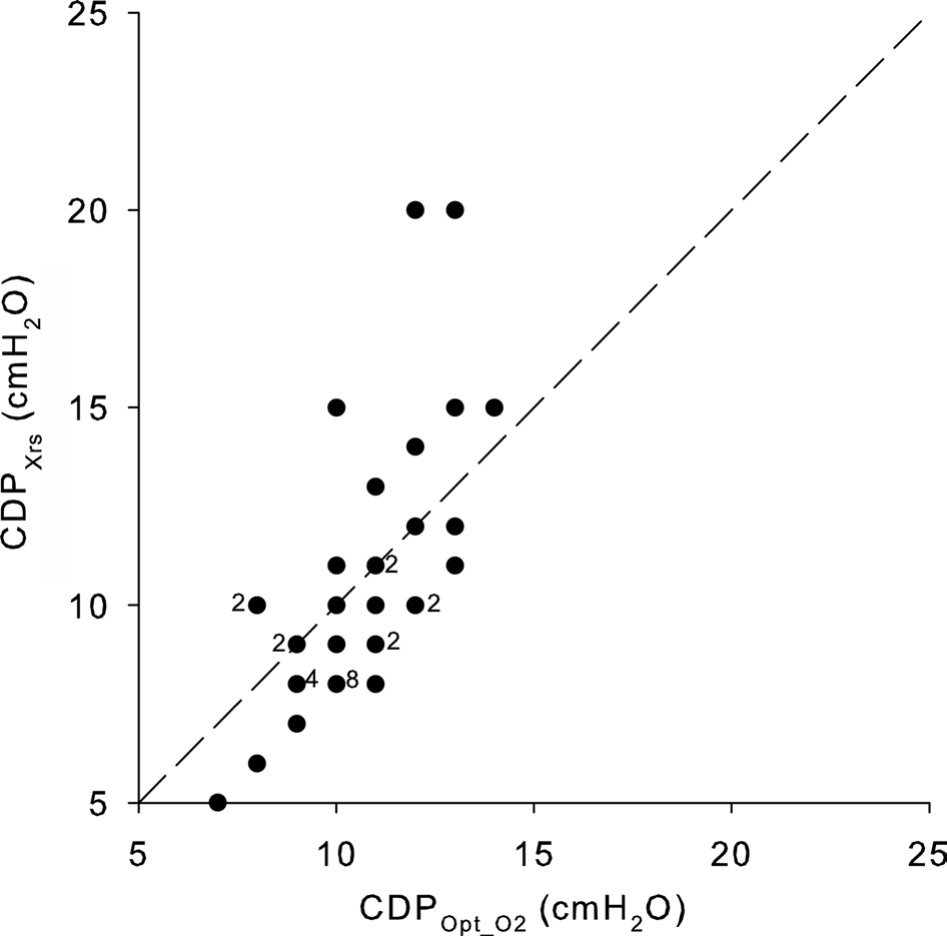
Comparison between CDP at the CDP corresponding to maximal Xrs (CDP_Xrs_) and the optimal CDP as identified by SpO_2_ (CDP_Opt_O2_). The number of subjects represented by each dot is reported for dots representing more than one infant

CDP_Opt_SpO2_ and CDP_Xrs_ were highly correlated (ρ = 0.71, p < 0.001) and not statistically different (median difference [range] = -1 [-3; 8]) (Fig. 3). CDP_Xrs_ were equal to CDP_Opt_SpO2_ in 6 infants (15%), greater than CDP_Opt_SpO2_ in 10 (25%), and lower in 24 infants (60%). The difference between CDP_Xrs_ and CDP_Opt_SpO2_ was greater than 2 cmH_2_O*s/L in only four subjects (Fig. 3, Additional file 1: Figure S2). However, the difference can be underestimated as CDP_Xrs_ matched CDP_min_ in 18 infants, where we did not identify an Xrs decrease. Xrs was higher at CDP_Xrs_ than at CDP_Opt_SpO2,_ while the other respiratory parameters were not different on average (Table 2).

### The overall effect of the procedure

CDP_f_ was not significantly different from CDP_i_ (Table 2). In contrast, Xrs and SpO_2_/FiO_2_ were higher and OSI lower after the maneuver, indicating successful lung recruitment. Changes in SpO_2_/FiO_2_ pre-post maneuver were poorly correlated with both CDP and Xrs changes (0.34, p = 0.03 and 0.33, p = 0.04, respectively).

## Discussion

In this study, we reported the changes in Xrs during recruitment maneuvers in a heterogeneous population of extremely preterm infants receiving HFOV. Our main findings were: (1) measuring Xrs at the bedside in patients receiving HFOV was feasible and provided a high percentage of good quality data; (2) Xrs changed significantly during the recruitment maneuvers. Its variable baseline values and variable changes with CDP indicated a heterogeneity of lung mechanical condition in ventilated preterm newborns; (3) the majority of the infants (87.5%) presented a significant hysteresis of the Xrs vs. CDP curve, suggesting that exposing the lung to the high CDP of the maneuver resulted in lung volume recruitment. The hysteresis of the Xrs vs. CDP curve was not entirely predictable by the Xrs changes during the inflation limb. Also, 55% of infants presented an Xrs decrease at the end of the deflation limb, indicating lung volume derecruitment occurring after 3 min at the lowest CPD applied. Re-recruitment after the maneuver restored Xrs values of the deflation limb at the corresponding CDP in 87.5% of maneuvers; (4) on average, changes in Xrs correlated with changes in SpO_2_/FiO_2_. However, Xrs mainly improved on the deflation limb, whereas SpO_2_/FiO_2_ improved primarily on the inflation limb. Even if CDP_Xrs_ and CDP_Opt_SpO2_ were not statistically different overall, they differed most of the time, indicating that CDP_Opt_SpO2_ did not always attain optimal lung mechanics.

This is the first study addressing Xrs changes with CDP in a large population of preterm infants receiving HFOV. Only a few previous studies [9, 15, 21] reported changes in Xrs with distending pressure in intubated preterm infants, and only one of them was performed during HFOV [9]. In our study, only 2% of measurements were unsuccessful, and only 3% of the data points were outliers, supporting the feasibility of oscillometry measurements in clinical settings. The wide ranges of the parameters describing Xrs vs. CDP curve (ΔX_inf_limb_, ΔX_cl_, and ΔX_rec_) highlight the heterogeneity of lung condition, distensibility, recruitability, and tendency to derecruitment in our population.

### Xrs vs. CPD curve: Xrs changes during the inflation limb and curve hysteresis

Figure 1 shows a marked decrease in Xrs with increasing CDP (ΔX_inf_limb_ >0). Xrs changes during the inflation limb are determined by the balance between lung volume recruitment and increased lung tissue distention. Lung volume recruitment increases Xrs, whereas increased lung tissue distension (i.e., reduced tissue compliance) lowers Xrs [22]. Steeper decreases may be associated with greater tissue stress [16]. However, ΔX_inf_limb_ was small or even negative in some infants, indicating that Xrs changes were mainly driven by lung volume recruitment. These data show that reaching the opening CDP according to SpO_2_ exposes the lung to very variable stress between infants.

ΔX_rec_ is related to the hysteresis in the Xrs vs. CDP graph and, comparing Xrs at the same CDP during inflation and deflation, provides an indication of the recruited lung, as the contribution to Xrs of tissue distention is the same at the same CDP. 87.5% of infants had ΔX_rec_ higher than the reproducibility of the Xrs measure, which testifies to lung volume recruitment. As 92.5% of these maneuvers were not performed at HFOV initiation, our results suggest that lung volume derecruitment may frequently occur during long periods of mechanical ventilation at constant CDP, and periodical CDP optimizations should be performed to maintain proper lung recruitment [23]. Also, 15 infants showing significant lung volume recruitment were on mechanical ventilation for over 6 days. This suggests that, in some patients, CDP optimizations can be effective also in a post-acute phase. Also, the general improvement of infant conditions after the overall procedure further supports the efficacy of the performed maneuvers.

However, ΔX_rec_ was variable and not predictable from the patterns of Xrs changes with CDP during the inflation limb (see Additional File 1). If and how Xrs changes during the inflation limb can be used to tailor recruitment maneuvers for improving safety and efficacy remains to be addressed. The increasing Xrs with time at fixed CDP (Fig. 2, right panel) suggests the progression of slow lung volume recruitment during some CDP steps of the inflation limb. Future studies should address this topic for understanding the optimal step duration [24]. Repeating oscillometry tests during each CDP step may provide criteria for tailoring the step duration by identifying the stability of Xrs.

### Xrs vs. CPD curve: last steps of the deflation limb and re-recruitment after the maneuver

Xrs changes during the deflation limb are determined by the balance between lung volume de-recruitment and decreased lung tissue distention. Lung volume derecruitment lowers Xrs, whereas reduced lung tissue distension increases Xrs [22]. The average behavior shows higher Xrs values at the lowest CDPs, indicating how easily the lung tissue is distended by pressure in preterm infants. However, in surfactant-depleted or collapsible lungs, the low CDPs reached at the end of the deflation limb can promote lung volume derecruitment and result in Xrs reduction with decreasing pressure toward the end of the maneuver (ΔX_cl_>0). Steeper decreases indicate greater lung periphery instability (i.e., a higher tendency to de-recruit). This characteristic of the Xrs vs. CDP loops was very variable between infants, with Xrs decreasing at the end of the maneuver in 55% of the infants.

We did not find previous studies addressing the efficacy of the second phase of the recruitment maneuver, which consists of reaching the opening CDP (CDP_max_) for a few minutes before setting CDP_Opt_O2_. Lung physiology suggests that this procedure should be performed after reaching CDP_min_, to reverse the de-recruitment provoking the SpO_2_ drop defining the closing CDP. In our population, this procedure restored the Xrs value reached at CDP_OPt_O2_ of the deflation limb in 87% of maneuvers. In a previous study during conventional ventilation [16], restoring the clinical set positive end-expiratory pressure (PEEP) directly after 5 min at the clinical PEEP – 2cmH_2_O without reaching the opening pressure resulted in persistent de-recruitment in 32% of infants. This finding underlines the importance of performing this procedure after each recruitment maneuver.

### Xrs and oxygenation changes

In our dataset, in accordance with previous studies [25, 26], oxygenation improved during inflation and remained more stable during deflation, whereas Xrs, deteriorated during inflation and improved during deflation. Oxygenation may be less sensitive to overdistension and less accurate in defining the optimal point of ventilation because of the uniformity of SpO_2_ readings over a wider range of airway pressures during the deflation limb. Similarly to what was reported for other respiratory variables studied during SpO_2_-driven maneuvers [27, 28], Xrs identified a range of optimal pressures just above CDP_min_, in line with the definition of CDP_Opt_O2_.

The CDP value corresponding to the maximal Xrs may provide the optimal mechanical balance between maximizing lung volume recruitment and reducing tissue overdistention. This CDP (CDP_Xrs_) correlates with CDP_Opt_O2_ but is not identical in 85% of the maneuvers, with a tendency to be lower, as previously noticed also during conventional ventilation [15]. Applying CDP_Xrs_ instead of CDP_Opt_O2_ would have led globally to higher Xrs, suggesting that Xrs may identify more protective ventilation settings. Future studies must clarify if this lower CDP setting can warrant similar gas exchange and stable lung mechanics in time.

We found a difference between CDP_Xrs_ and CDP_Opt_SpO2_ greater than 2 cmH_2_O*s/L in only four subjects (Fig. 3 and Additional File 1: Figure S1). Infants with CDP_Xrs_ higher than CDP_Opt_O2_ would likely have to deal with insufficient pressure to optimize lung mechanics and recruitment, leading to possible increased work of breathing and/or lung tissue stress. A higher CDP_Xrs_ than CDP_Opt_O2_ can be due to different factors. First, Xrs was measured 3 min after the CDP change, while SpO_2_ after a longer period. Xrs may still be decreasing after 3 min at the lowest CDPs as slow lung derecruitment may occur [29]. Second, Xrs may not identify increasing inhomogeneity due to a small volume loss until significant lung derecruitment occurs [30].

Determining CDP_Xrs_ can improve CDP tailoring as it increases the awareness of the lung mechanical conditions and, therefore, the risk of tissue stress and lung derecruitment.

### Limitations

This study has some limitations. Xrs was measured 3 min after the CDP change as by default ventilator setting while SpO_2_ was recorded at the end of each CDP step (in median 6 min after CDP change) when infant conditions were judged stable. Therefore, SpO_2_ and Xrs values are compared at different time points. Also, CDP steps of 2 cmH_2_O during the deflation limb limited our resolution in comparing CPD_opt_SpO2_ and CDP_Xrs_. Finally, performing oscillometry required a short interruption of HFOV. However, as the procedure lasted only 3–5 s and the CDP was not changed, it should not have significantly affected lung mechanics and gas exchange.

In conclusion, we described Xrs changes during SpO_2_-guided recruitment maneuvers in extremely preterm infants. The Xrs vs. CDP loop provides information on the lung mechanical status, which is complementary to oxygenation and may allow for improving the individualization of ventilatory settings at the bedside during HFOV. Further investigation is warranted to evaluate the impact of tailoring recruitment maneuvers and CDP according to Xrs measures on short- and long-term respiratory outcomes.

### Electronic supplementary material

Below is the link to the electronic supplementary material.

Additional file 1. Description of Xrs vs. CPD curve patterns during the inflation limb and resulting curve hysteresis. Description of the CDP trials of the four infants with the highest difference between CDP_Opt_SpO2_ and CDP_Xrs_.

## Data Availability

The datasets used and/or analysed during the current study are available from the corresponding author on reasonable request.

## List of abbreviations

BPD: bronchopulmonary dysplasia
CDP: continuous distending pressure
CDP_f_: final CDP at the end of the procedure, after re-recruiment after the deflation limb
CDP_i_: initial CDP of the maneuver
CDP_max_: maximal CDP of the maneuver
CDP_min_: minimal CDP of the maneuver
CDP_Opt_SpO2_: optimal CDP as identified by peripheral oxygen saturation
CDP_Xrs_: the CDP providing maximal Xrs on the deflation limb of the recruitment maneuver
FiO_2_: fraction of inspired oxygen
GA: gestational age
HFOV: high-frequency oscillatory ventilation
OSI: oxygen saturation index
SpO_2_: peripheral oxygen saturation
TcCO_2_: transcutaneous CO_2_
Xrs: respiratory reactance
ΔX_inf_limb_: change in Xrs at the end of the inflation limb, computed as the difference in Xrs between CDP_max_ and CDP_i_
ΔX_cl_: decrease in Xrs at the closing CDP (CDP_min_) from its maximal value (at CDP_Xrs_)
ΔX_rec_: change in Xrs associated with possible lung volume recruitment that occurred during the maneuver, computed as the difference in Xrs at CDP_Xrs_ during the deflation and the inflation limbs
