# Supplementary material for: Oscillometry for personalizing continuous distending pressure maneuvers: an observational study in extremely preterm infants
Source: Respir Res. 2024 Jan 4;25:4. doi: 10.1186/s12931-023-02639-4 (PMC10765834; doi:10.1186/s12931-023-02639-4)
Supplement: Supplementary file 1 — Additional file 1. Description of Xrs vs. CPD curve patterns during the inflation limb and resulting curve hysteresis. Description of the CDP trials of the four infants with the highest difference between CDPOpt_SpO2 and CDPXrs. [file 12931_2023_2639_MOESM1_ESM.docx]

**Oscillometry for personalizing continuous distending pressure maneuvers: an observational study in extremely preterm infants**

Chiara Veneroni^1^, Raffaele L. Dellaca'^1^, Erik Küng^2^, Beatrice Bonomi^1^, Angelika Berger^2^

Tobias Werther^2^

^1^ TechRes Lab, Department of Electronics, Information and Biomedical Engineering (DEIB), Politecnico di Milano University, Milan, Italy

^2^ Division of Neonatology, Pediatric Intensive Care and Neuropediatrics, Department of Pediatrics, Comprehensive Center for Pediatrics, Medical University of Vienna, Vienna, Austria

**ORCID ID**: Chiara Veneroni http://orcid.org/0000-0002-8607-1728

Raffaele L. Dellacà http://orcid.org/0000-0002-3672-4591

**Correspondence:** Raffaele L. Dellacà Dipartimento di Elettronica, Informatica e Bioingegneria, Politecnico di Milano, Via G. Colombo 40, Milano -20133, Italy. Email: raffaele.dellaca@polimi.it. Tel: +39 02 2399 9024

**Patterns of Xrs vs. CPD curve during the inflation limb and resulting curve hysteresis**

We identified 4 different groups (Figure S1) with different patterns: group A (n=8, 20%) Xrs increased during the inflation limb and ΔX_rec_ > 6.3 cmH_2_O*s/L and 10%; group B (n=5, 12,5%) Xrs decreased during the inflation limb and ΔX_rec_ < 6.3 cmH_2_O*s/L and 10%; group C (n=14, 35%) Xrs decreased during the inflation limb but ΔX_rec_ > 6.3 cmH_2_O*s/L and 10%; and group D (n=12, 30%) Xrs decrease during the inflation limb except for at least 2 steps in which Xrs was stable despite increasing CDP and ΔX_rec_ > 6.3 cmH_2_O*s/L and 10%. We did not find statistically significant differences in the groups' infant demographics/clinical parameters.

The first pattern (group A) is similar to the one reported in studies on surfactant-depletion experimental models (1,2). It is characterized by an initial Xrs increase with increasing CDP as lung recruitment occurs, followed by an Xrs decrease or stability when lung and chest wall tissue distension overcomes the contribution of lung volume recruitment. All recruitment maneuvers of this group showed a significant hysteresis of the Xrs vs. CDP curve, confirming lung volume recruitment resulting from the maneuvers. The second pattern (group B) resembles the one reported in a previous study on newborns during HFOV (3). It is characterized by decreasing Xrs with increasing CDP ascribed to the prevalence of tissue distention over recruitment during the inflation limb in line with the absence of significant Xrs vs. CDP curve hysteresis. The third pattern (group C) resembles the one reported for low birth weight RDS infants during conventional ventilation (4). Despite Xrs consistently decreasing with increasing CDP, the Xrs vs. CDP curve showed significant hysteresis with less negative Xrs measured on the deflation limb, indicating the occurrence of lung volume recruitment. The last pattern (Group D), characterized by Xrs decrease followed by stability for more than two CPD steps during the inflation limb, was not reported before. Patterns of groups C and D may result from slower lung volume recruitment during the inflation limb, as suggested by Figure 2, right panel, where Xrs increased with time at fixed CDPs in a subject of group C. Future studies should clarify this topic. Repeating oscillometry measures at a constant CDP may provide a criterion for tailoring the step duration at a given CDP by identifying the stability of oscillatory mechanics.

*
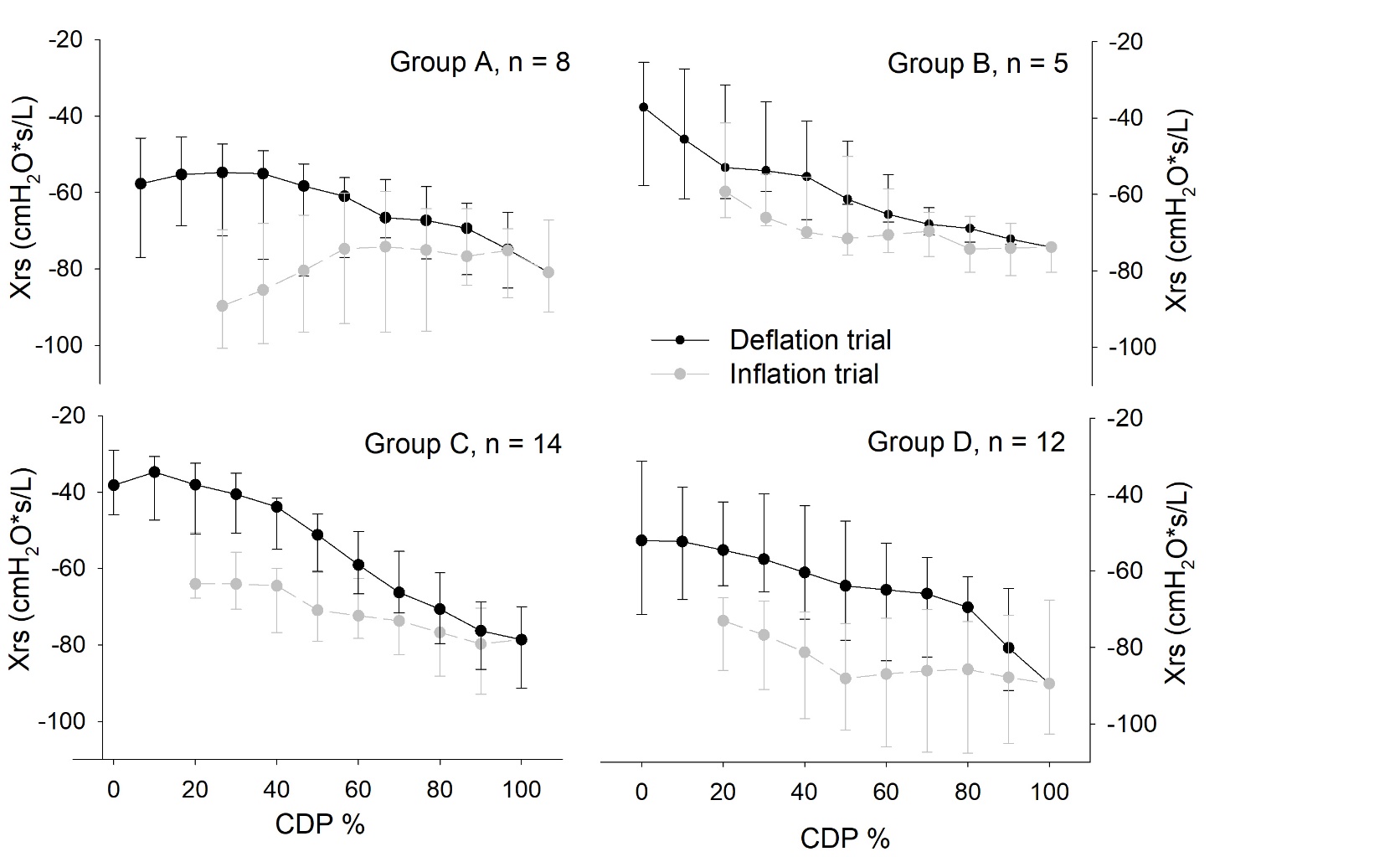
*

**Figure S1.** Different Xrs vs. CDP patterns. One infant was excluded from this analysis as the missing Xrs values during the inflation limb prevented the classification.

**Infants with different CDP_Opt_SpO2_ and CDP_Xrs_**

Three infants presented CDP_Xrs_ more than 2 cmH_2_O*s/L higher than CDP_Opt_O2_. These infants may have to deal with insufficient pressure to optimize lung mechanics and recruitment, leading to possible increased work of breathing and/or lung tissue stress. This may be the case for infants #1 and #2 of Figure S2. SpO_2_ was slowly decreasing with decreasing CDP also above CDP_Opt_O2_ although not reaching the clinical threshold. Infant #1 was suffering from necrotizing enterocolitis (large abdomen, diaphragm pushed cranially, leading to reduced functional residual capacity), where larger CDP levels may be needed. Approx. 2 hours after this maneuver, the FiO_2_ needed to be raised to 50%, supporting the idea that the chosen CDP according to SpO_2_ was too low. Infant #3 needed very low FiO_2_, which may increase difficulties in determining an optimal CDP for oxygenation. Conversely, the higher CDP_Xrs_ than CDP_Opt_O2_ in infant #2 resulted from stable Xrs values during the deflation limb: Xrs decrease was <1 cmH_2_O*s/L over 6 cmH_2_O of CDP reduction.

One infant (#4) presented CDP_Xrs_ more than 2 cmH_2_O*s/L lower than CDP_Opt_O2_. As reported in the manuscript, this difference may be ascribed to 1) Xrs being measured before reaching lung mechanical stability and SpO_2_ being evaluated later or 2) low Xrs sensitivity to small volume loss that can increase inhomogeneity of ventilation (5).

**
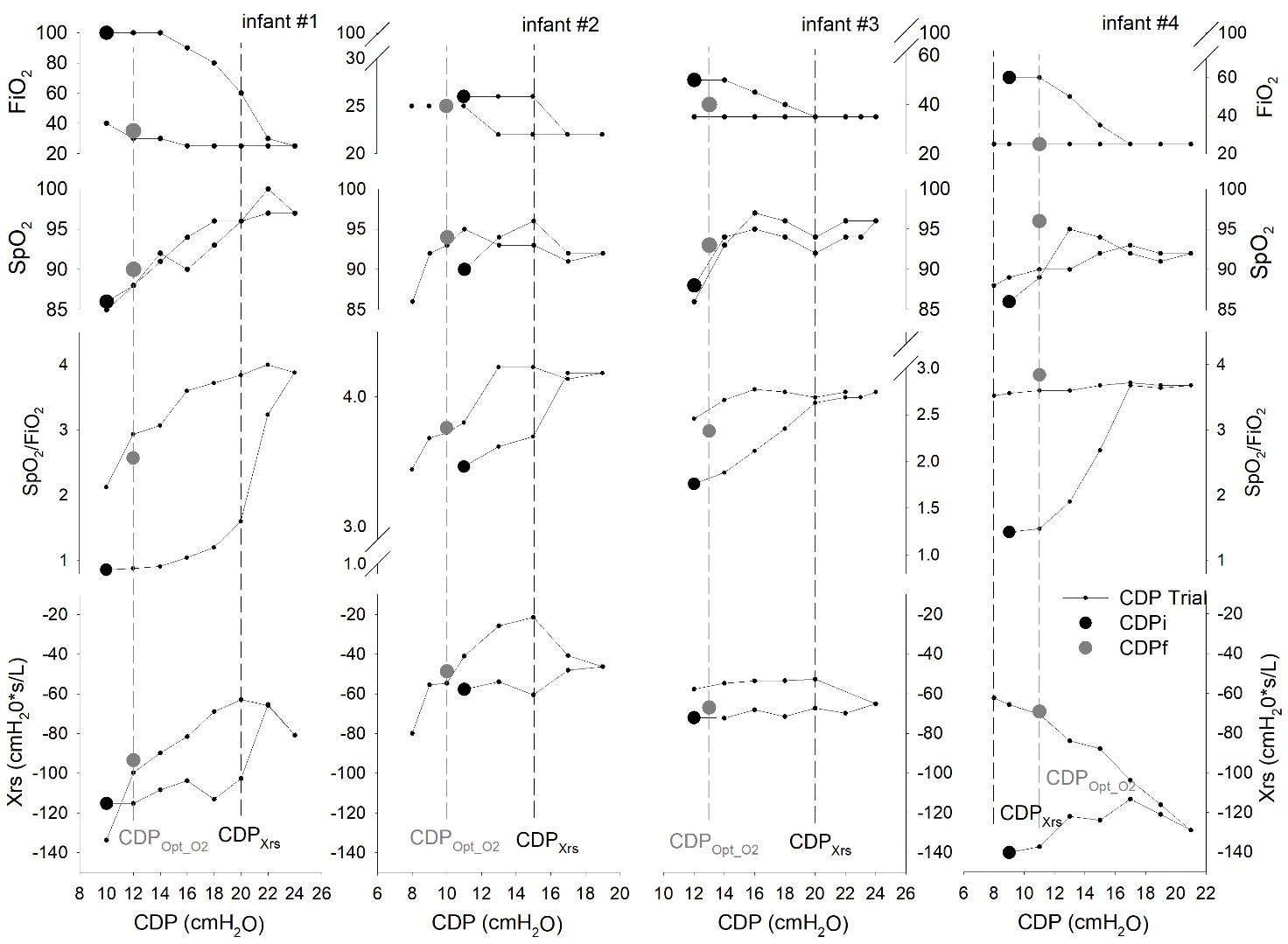
**

**Figure S2.** Changes in Xrs, FiO_2_, SpO_2_, and SpO_2_/FiO_2_ during the recruitment maneuver for the four subjects having a difference > 2 cmH_2_O between CDP_Xrs_ and CDP_Opt_O2_. Dashed black and grey lines identify CDP_Xrs_ and CDP_Opt_O2_, respectively. Black and grey dots identify the initial CDP (CDPi) and the final CDP after re-recruitment (CDP_f_), respectively.

**References**

1. Dellacà RL, Zannin E, Kostic P, Olerud MA, Pompilio PP, Hedenstierna G, et al. Optimisation of positive end-expiratory pressure by forced oscillation technique in a lavage model of acute lung injury. Intensive Care Med. 2011 Jun;37(6):1021–30.

2. Dellacà RL, Zannin E, Ventura ML, Sancini G, Pedotti A, Tagliabue P, et al. Assessment of Dynamic Mechanical Properties of the Respiratory System During High-Frequency Oscillatory Ventilation. Crit Care Med. 2013 Jun 10;

3. Zannin E, Doni D, Ventura ML, Fedeli T, Rigotti C, Dellacá RL, et al. Relationship between Mean Airways Pressure, Lung Mechanics, and Right Ventricular Output during High-Frequency Oscillatory Ventilation in Infants. J Pediatr. 2017;180:110–5.

4. Dellacà RL, Veneroni C, Vendettuoli V, Zannin E, Matassa PG, Pedotti A, et al. Relationship between respiratory impedance and positive end-expiratory pressure in mechanically ventilated neonates. Intensive Care Med. 2013 Mar;39(3):511–9.

5. Veneroni C, Van Muylem A, Malinovschi A, Michils A, Dellaca RL. Closing volume detection by single-breath gas washout and forced oscillation technique. J Appl Physiol. 2021;130(4):903–13.
